# Early Postnatal Lipopolysaccharide Exposure Leads to Enhanced Neurogenesis and Impaired Communicative Functions in Rats

**DOI:** 10.1371/journal.pone.0164403

**Published:** 2016-10-10

**Authors:** Yi Pang, Xuemei Dai, Anna Roller, Kathleen Carter, Ian Paul, Abhay J. Bhatt, Rick C. S. Lin, Lir-Wan Fan

**Affiliations:** 1 Department of Pediatrics, University of Mississippi Medical Center, Jackson, Mississippi, United States of America; 2 Department of Psychiatry and Human Behavior, University of Mississippi Medical Center, Jackson, Mississippi, United States of America; 3 Department of Neurobiology and Anatomical Sciences, University of Mississippi Medical Center, Jackson, Mississippi, United States of America; Hopital Robert Debre, FRANCE

## Abstract

Perinatal infection is a well-identified risk factor for a number of neurodevelopmental disorders, including brain white matter injury (WMI) and Autism Spectrum Disorders (ASD). The underlying mechanisms by which early life inflammatory events cause aberrant neural, cytoarchitectural, and network organization, remain elusive. This study is aimed to investigate how systemic lipopolysaccharide (LPS)-induced neuroinflammation affects microglia phenotypes and early neural developmental events in rats. We show here that LPS exposure at early postnatal day 3 leads to a robust microglia activation which is characterized with mixed microglial proinflammatory (M1) and anti-inflammatory (M2) phenotypes. More specifically, we found that microglial M1 markers iNOS and MHC-II were induced at relatively low levels in a regionally restricted manner, whereas M2 markers CD206 and TGFβ were strongly upregulated in a sub-set of activated microglia in multiple white and gray matter structures. This unique microglial response was associated with a marked decrease in naturally occurring apoptosis, but an increase in cell proliferation in the subventricular zone (SVZ) and the dentate gyrus (DG) of hippocampus. LPS exposure also leads to a significant increase in oligodendrocyte lineage population without causing discernible hypermyelination. Moreover, LPS-exposed rats exhibited significant impairments in communicative and cognitive functions. These findings suggest a possible role of M2-like microglial activation in abnormal neural development that may underlie ASD-like behavioral impairments.

## Introduction

Very low birth weight infants (VLBW, <1500 g) are at great risk for developing long-term neurological disabilities [1]. Decades ago, Periventricular Leukomalacia (PVL), which is a necrotic form of brain white matter injury, was the predominant cause of neurological morbidity in this group. Thanks to markedly improved neonatal care, the survival rate of VLBW infants has been greatly improved. Unfortunately, many of those survivors live with neurological disabilities that manifest predominantly as non-motor related symptoms, ranging from sensory, cognitive, attentional, language, executive, to behavioral impairments [2, 3]. Some of those behavioral problems such as impairments in language, communication and social behaviors, are among the core behavioral symptoms of Autism Spectrum Disorders (ASD), which is a pervasive neurodevelopment disorder with no clearly defined etiology and neuropathology. Epidemiological studies suggest that the prevalence of ASD among VLBW infants is approximately 5-fold higher than term infants [4, 5]. Given that perinatal infection has been strongly linked to the etiology of both WMI and ASD [6, 7], it is possible that there may be shared underlying pathophysiology between these two groups, at least in a subset of patients.

The diffuse WMI, which is characterized by microscopic damage to developing oligodendrocytes (OLs) and axons in the white matter track, is now more common than PVL. Although the diffuse WMI might be chiefly responsible for neurological/neurobehavioral deficits in affected VLBW infants [8], MRI confirmed cases account for only one-third of all neurologically impaired patients [9, 10], leaving the majority of neurologically/behaviorally impaired very premature infants without clear evidence of brain injury. This suggests that abnormal growth in gray matter structures may also contribute to neurological morbidity of those surviving infants, especially concerning cognitive and neurobehavioral impairments. Recently, the term “encephalopathy of prematurity” has been introduced to highlight the importance of other brain areas beyond the white matter [8].

Since perinatal infection/inflammation has been identified as a major risk factor for WMI by a large body of epidemiological studies [6, 11, 12], a number of infection/inflammation-based animal models (mostly in rodents) have been developed to study the underlying mechanisms of this disorder. The most commonly used approach is to treat animals with bacterial endotoxin, lipopolysaccharide (LPS), for initiating neuroinflammatory response via either intracerebral or systemic applications. In our previous studies, we have demonstrated that intracerebral injection of LPS to postnatal rats induces typical neuropathological features of PVL, including periventricular white matter lesion, ventriculomegaly, and myelination impairments [13–15]. At the cellular level, intracerebral LPS injection induces a robust microglia activation and subsequent proinflammatory cytokine release, which are associated with death of OL progenitor cells, disturbances in OL development, as well as axonal injury [13, 14, 16]. The limitation of this model, however, is that the route of LPS administration is less clinically relevant given that majority of perinatal infection are maternal or systemic in origin. The decreased incidence of PVL (less than 5% nowadays) and increased encephalopathy of immaturity including diffuse WMI [17], calls for developing more clinically relevant animal models to study the mechanisms underlying aberrant brain development especially those pertinent to cognitive and behavioral impairments. Therefore, the current study is aimed to test the hypothesis that systemic LPS exposure during early postnatal period may lead to a less severe forms of WMI (e.g. diffuse WMI) and/or abnormal gray matter growth, reflecting cognitive and behavioral deficits in rats. Interestingly, our data show that systemic LPS does not induce neural injury, but instead leads to a hypertrophic effect on neural development. Moreover, neurodevelopmental abnormalities are associated with ASD-like neurobehavioral dysfunctions in rats.

## Material and Methods

### Ethics Statement

This study was conducted in strict accordance with the National Institutes of Health Guide for the Care and Use of Laboratory Animals. The study was approved by the Institutional Animal Care and Use Committee at the University of Mississippi Medical Center. All efforts were made to minimize the discomfort and stress of animals.

### Chemicals and Reagents

Unless otherwise stated, all chemicals used in this study were purchased from Sigma (St. Louis, MO, USA). The sources of kits, antibodies, and other reagents are listed below: Terminal deoxynucleotidyl transferase mediated dUTP nick end labeling (TUNEL) kit: Millipore (Billerica, MA, USA). Western blot reagents (Life Technologies, Grand Island, NY, USA). ECL select kit: GE healthcare (Piscataway, NJ, USA). Antibodies used are list in Table 1:

**Table 1:** Summary of antibodies used in this study.

*Antibodies*	*Source*	*Cat#*	*Target(s) of labeling*
Myelin basic protein (MBP)	Millipore	MAB381	myelin
PDGF receptor-α (PDGFR)	Santa Cruz	Sc-9974	OL progenitor cells (OPC)
OL transcription factor 2 (Olig2)	Millipore	AB9610	total OL lineage (nucleus)
Adenomatous Polyposis Coli (APC)	Millipore	OP80	mature OLs (cell body)
CD11b (OX-42)	Millipore	CBL1512	microglia/macrophage
ED1	Millipore	MAB1435	activated microglia/macrophage
Ionized calcium binding adaptor molecule 1 (Iba1)	Wako Lab	019–19741	microglia/macrophage
Inducible nitric oxide synthase (iNOS)	Chemicon	AB5382	M1 marker
Major histocompatibility complex-II (MHC-II)	USbiological	M3887-10B	M1 marker
CD206	Abcam	Ab8918	M2 marker
Transforming growth factor beta (TGFβ)	Abcam	Ab66043	M2 marker
pSmad3	Cell Signaling	12747	total Smad3
Smad 3	Cell Signaling	12747	total Smad3
Caspase-3 (cleaved)	Cell Signaling	9664	active form of caspase-3
Doublecortin (Dcx)	Cell Signaling	4604	neuroblasts

### Animal Treatments

Time-pregnant Sprague-Dawley rats were purchased from Harlan Laboratories (Indianapolis, IN). Animals arrived in the Laboratory Animal Facility (LAF) on day 18 of gestation and gave birth on day 22. Both male and female offspring were used in this study.

The day of birth was defined as postnatal day 0 (P0). On P3, pups (both males and females) were intraperitoneally (i.p.) injected with LPS (from E. coli, serotype O55:B05, Sigma-Aldrich, MO) at 1 mg/kg body weight. This dose of LPS is equivalent to what we have used in the intracerebral LPS model [13]. Control rats received the same volume of sterile saline solution (100 μl). After injection, pups were returned to their nursing dam. Numbers of animals per litter were adjusted to 10. Rat pups were weaned on P21.

### TUNEL and Immunohistochemistry

On P6, P12 and P21, rats were transcardially perfused with normal saline followed by 4% paraformaldehyde (PFA). Rat brains were post-fixed in 4% PFA for 48 h, followed by incubation in sucrose solutions (sequentially in 10%, 20%, and 30%, each for at least 24 h) for cytoprotection. Free-floating coronal brain sections (40 μm) were prepared using a freezing microtome (Leica, SM 2000R, Wetzlar, Germany). TUNEL staining was performed following manufacture’s instruction, with the exception that the sections were pre-treated with 0.5% triton (in PBS) for 1 h at RT to facilitate TDT enzyme penetrating into nuclei. For immunostaining, sections were first washed with PBS, blocked with 10% normal goat serum (Millipore) in PBS for 1 h at room temperature (RT), and then incubated with primary antibodies overnight at 4°C. The next day, sections were washed with PBS and then incubated with secondary antibodies conjugated with Alex fluo488 (1:300) or 555 (1:2000) at RT for 1 h. Sections were then washed and mounted on slides. DAPI (100 nM) was included in the mounting medium for counter-staining. Sections were viewed under a fluorescence microscope (Nikon NIE, Nikon Instruments Inc., Melville, NY, USA) and images were acquired by Nikon Nis Element software.

### Cell Counting

All cell counting was conducted by ImageJ software using the automatic cell counting function, as described previously [15, 18]. Since TUNEL+ cells were found in highest density in the caudate putamen (CPu), they were counted in this region. Three images were captured for a single section using 25× objective by a monoclonal digital camera, and 3 consecutive sections were included in final analysis representing as a single brain sample. Iba1+ cells were counted in the hippocampus in a similar manner. For PDGFR+, APC+ and Olig2+ cell counting, 5 adjacent images at the corpus callosum were captured under 40x objective. Ki67+ cells were counted in the dentate gyrus (DG), while the areas occupied by ki67 immunostaining in the subventricular (SVZ) were determined by the ImageJ software. A total of 8 animals were included in each treatment.

### Immunoblotting

On P6, rats were sacrificed for dissecting brain tissue. Total proteins were extracted using the tissue lysis buffer (Invitrogen) supplemented with protease inhibitor cocktails (Sigma). Total protein contents in the lysate were determined by the BCA method, and were subsequently adjusted at 1 mg/ml. Samples were denatured and subjected to SDS-PAGE, and proteins were transferred to nitrocellulose membranes. For immunoblotting, the membranes were first blocked with 5% non-fat milk/1% BSA in PBS for 2 hr at RT and then incubated with anti- TGFβ or pSmad antibodies overnight at 4°C. Following washing, membranes were incubated with horseradish peroxidase-conjugated second antibody, and signals were detected using the ECL select system. After blotting for each of the target proteins, the membranes were stripped and re-probed for alpha-tubulin or non-phosphorylated Smad3 as the loading controls. Images were acquired by ChemiDoc MP Imaging system and data were analyzed by Image Lab software (Bio-Rad).

### Behavioral Tests

Behavioral tests were conducted in the Animal Behavior Core. The experimenter conducting the behavioral tests was unware of the treatment conditions.

### Ultrasonic Vocalization

This test is based on the findings that rats communicate vocally to conspecifics using ultrasonic vocalizations in the range between 10–100 kHz [19]. For rat pups, brief isolation from their dams caused them to emit a characteristic 35–40 KHz calls, and this behavior can be used to assess the development of basic social communication [20]. Briefly, rat pups at P10 were separated from their dams to a holding room. The vocalizations were recorded using the Metris Sonotrack USV detection system (Hoofddorp, the Netherlands). Pups were recorded individually for 120 sec and then returned to their dam. Data were analyzed using the Sonotrak system (Metris).

### Novel Object Recognition Test

This test is used to evaluate exploratory and cognitive functions in rodents. Animals were placed in a locomotor activity monitoring chamber (Automex, Columbus Instruments, Columbus, OH) with the size of 42×42 cm, and their activity was monitored by infrared sensors that divided the chamber into 16 zones. On P40, animals were allowed to explore the chamber for 20 min, and a novel object (white, Nalgene-covered block) was then placed in a randomly chosen corner and the rats were monitored for another 10 min. Exploration of the four corner zones (entries in square, and duration in square) was compared between the first 10 min exploration epoch (baseline) and the third 10 min exploration epoch (Novel object). Data were analyzed by one-way ANOVA.

### Data Analysis

Data were analyzed by unpaired t-test or one-way ANOVA (Novel object recognition test) using Sigma Plot software (version 12). All data were presented as Mean±SEM. A value of p<0.05 was considered statistically significant.

## Results

### Systemic LPS Exposure Leads to a Robust Microglial Activation Characterized with Both M1 and M2-Like Polarizations

**Fig 1:**
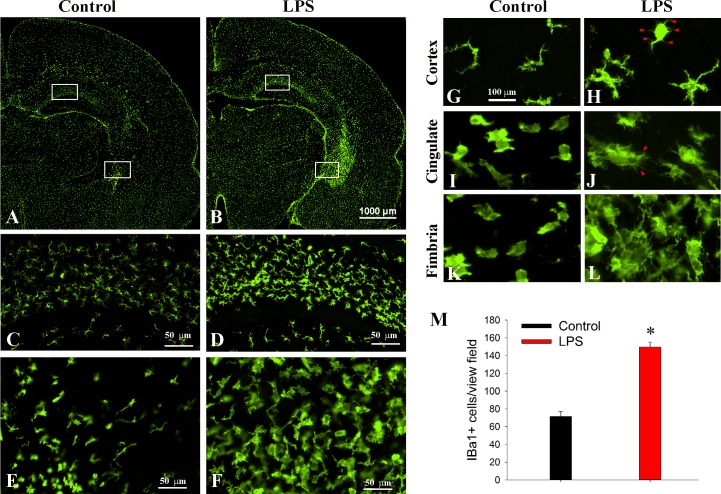
Morphological characteristics of activated microglia following systemic LPS exposure. A&B: hemispheric view of Iba1 immunostaining in coronal sections at the dorsal hippocampal level at P6. Compared to the control (A), a marked increase in Iba1+ cells was clearly evident in LPS-treated brain with regional heterogeneity. The most affected regions are the hippocampus and the major white matter tracks including the internal capsule (B). Enlarged areas in the white boxes at the hippocampus and internal capsule of the control and LPS treatment are shown in C/E and D/F, respectively. Higher magnification micrographs in G-L show typical Iba1+ cells in the cortex, cingulum, and fimbria of hippocampus in the control and LPS treatment. Note that except for larger cell bodies and thicker processes, a subset of Iba1+ cells in the LPS group exhibit much more numerous and finer processes (red arrows in H&J) as compared to the control. Cell counting in the hippocampus showed that the number of Iba1+ cells was more than doubled by LPS treatment (M). *p<0.001 vs control (n = 8).

**Fig 2:**
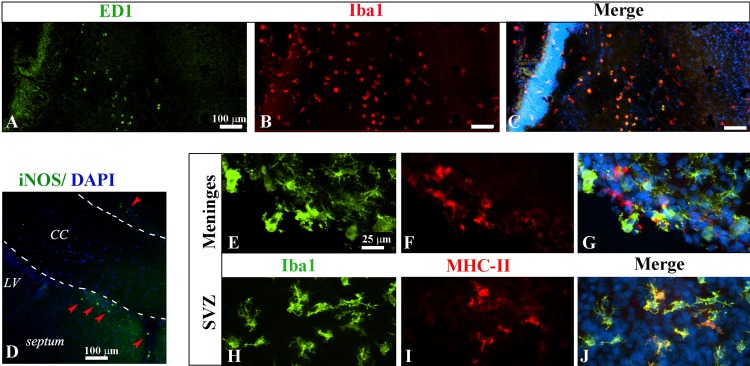
Proinflammatory markers are marginally induced in LPS-treated brain on P6. ED1+ cells were found in the major white matter tracks including the internal capsule, and they were co-localized with a subpopulation of IBa1+ cells (A-C). Very few iNOS+ cells were noted only in the meninges just above and underneath the corpus callosum (CC) (arrow heads in D). A small number of MHC-II+ cells were detected in the meninges overlaying the cerebral cortex (Fig 2E–2G), and the SVZ (H-J). *p<0.01 vs control (n = 8).

First we examined general morphological characteristics of microglia 3 days (on P6) following LPS treatment. As shown in Fig 1, LPS treatment led to a marked increase in Iba1+ microglial population as well as morphological transformation. Although the effect of LPS on microglia activation appeared to be global, there was a regional heterogeneity. Areas with the most significant changes include the white matter tracks (such as corpus callosum, cingulum, internal capsule, fornix, and the fimbria of hippocampus), the periventricular areas (SVZ and intrastriatal VZ), and the hippocampal formation, which was clearly noted at low magnification (Fig 1B). In the control, typical microglia in the cortex exhibited smaller and elongated cell bodies with a few long processes (Fig 1G), although they appeared to be much less ramified compared to typical microglia in the adult animals. A small population of amoeboid-like microglia was observed in major white matters such as the cingulum (Fig 1I), and fimbria of hippocampus (Fig 1K). In contrast, most Iba+ microglia in LPS treated rats exhibited activated morphology, characterized with larger amoeboid-like, or rod-shaped somata (Fig 1D, 1F, 1H, 1J and 1L). A subset of Iba1+ cells appeared to have numerous thin processes/filopodia (arrow heads, Fig 1H & 1J). This type of activated microglia morphology was not typically noted in the adult brain, suggesting they might be functionally distinct from the classically activated phenotype. Cell counting in the hippocampus area showed that the overall number of Iba+ cells was more than doubled by LPS treatment (Fig 1M). Although the functional diversity of activated microglia has been increasingly recognized in a number of neurodegenerative disorders [21], much less is known for the developing brain. To assess functional states of activated microglia in the LPS-treated rat brain, several M1 and M2 markers were double-immunolabeled with pan-microglia markers CD11b or Iba1 at P6. As shown in Fig 2, the classically activated M1 markers were only marginally detected in the LPS group, since either their expression levels or the number of positively labeled microglia were relatively low. For instance, iNOS+ cells were noted only in the meninges between the corpus callosum and the septum or the cortex (Fig 2D, arrow heads), while a small number of MHC-II+ cells were detected in the SVZ and meninges (Fig 2E–2J), but not other brain regions. The M1 markers were not detected in any of the controls. As a general marker for activated microglia, ED1+ was expressed by a subset of Iba1+ cells in the periventricular areas and major white matter tracks such as the internal capsule (Fig 2A–2C). Those amoeboid-like cells, however, were also noticed in the control, suggesting they are development-specific rather than an indication of activation.

**Fig 3:**
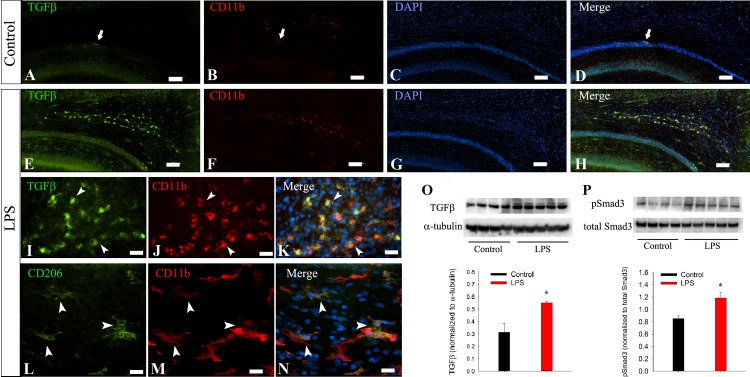
Systemic LPS treatment induces an M2-like microglial polarization on P6. Only a few weakly immunostained TGFβ +/CD11b+ cells could be noted in the subcortical white matter (A-D) of the controls. In contrast, TGFβ expression was strongly induced in amoeboid-like microglia by LPS treatment (E-H). High magnification micrographs (I-K) indicate exclusive co-localization of TGFβ with CD11b+ cells in the cingulum area. Although less numerous than TGFβ+ cells, CD206+/CD11b+ cells were observed in both the white matter and gray matter such as the thalamus (L-N) of LPS treated, but not the control rats. Immunoblotting showed that TGFβ (O) and pSmad3 levels were significantly increased by LPS treatment. *p<0.01 vs control (n = 8).

In contrast to M1 markers, several M2 markers were detected at relatively higher levels. For the controls, TGFβ immunostaining was mainly detected in neurons (data not shown), with a few scattered TGFβ +/CD11b+ cells noted in the white matter such as the corpus callosum (Fig 3A–3D, arrow). LPS induced a strong TGFβ expression in CD11b+ amoeboid-shaped microglia, which were clearly visible in subcortical white matter under low magnification (Fig 3E–3H). At a higher magnification, most TGFβ cells were found to be co-localized with CD11b+ cells (Fig 3I–3K). Similarly, numerous CD206+/CD11b+ microglia were observed in both the white and the gray matter (Fig 3L–3N, shown in the thalamus). Immunoblotting showed that TGFβ and its downstream signaling protein phosphorylated Smad3 (pSmad3) were significantly increased by LPS treatment (Fig 3O & 3P).

These M1 or M2 markers were no longer detectable on P21. However, based on morphological criteria, it appears that microglia from the LPS treated rats were not fully resolved at this stage. For example, the majority of control microglia within the cerebral cortex adopted ramified morphology, which is characterized with smaller soma and numerous, longer processes, as compared to microglia on P6. In contrast, significant more microglia in the LPS-exposed rats still exhibited larger cell bodies with less and shorter processes in similar regions. The difference in morphological characteristics were more pronounced in the white matter tracks and hippocampus (S1 Fig).

### LPS Exposure Markedly Suppresses Programmed Cell Death

**Fig 4:**
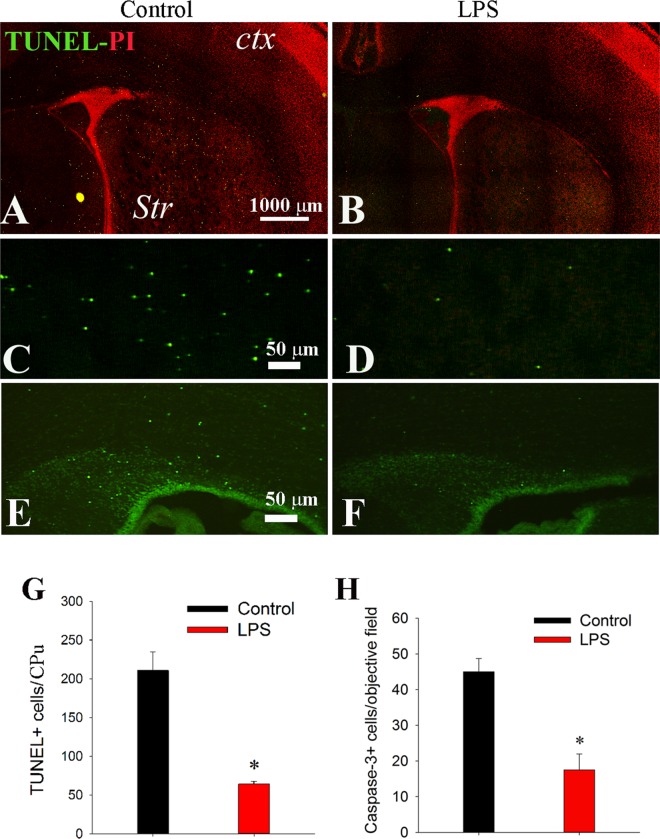
LPS exposure leads to a significant decrease in PCD on P6. Apoptotic cells were detected by TUNEL staining (green). A coronal section at the striatal level shows extensive TUNEL+ cells in the CPu, septum, SVZ, and periventricular white matter from a control rat (A). In contrast, significant fewer TUNEL+ cells were presented in the LPS treatment (B). Nuclei were counterstained with propidium iodide (PI, red). C and D: higher magnification micrographs of TUNEL staining in the CPu area from the control and LPS. E and F: immunostaining of cleaved caspase-3 in the periventricular area of the control and LPS. G and H: Quantification of TUNEL+ and caspase-3+ cells in the Cpu. *p<0.01 vs control (n = 8).

Apoptotic or programmed cell death (PCD) plays a critical role during normal neurodevelopment by serving as a refining mechanism to regulate neuronal [22] and oligodendroglial [23] numbers, while neuronal death induced by pathological insults such as hypoxia-ischemia or inflammation also manifested as a form of apoptosis. Therefore, we next investigated whether LPS exposure could increase PCD. In agreement with early reports that PCD peaks in the first postnatal week of rats [24], extensive apoptotic cell death was detected in the brain of control rats on P6. As shown in Fig 4, a large number of TUNEL+ cells were observed in the subcortical gray matter, including the CPu, septum, amygdala, thalamus, hypothalamus, etc., whereas fewer TUNEL+ cells were found in the cortex. Unexpectedly, LPS treatment led to a marked decrease in TUNEL+ cells. Cell counting in the CPu demonstrated that the number of TUNEL+ cells was reduced more than 3-fold by LPS exposure (Fig 4G). To verify the specificity of TUNEL for PCD, immunostaining of cleaved caspase-3 was conducted. Consistent with TUNEL data, the number of caspase-3+ cells was also significantly lower in LPS treatment (Fig 4H).

### LPS Exposure Leads to Over-Production of OLs without Affecting Myelination

**Fig 5:**
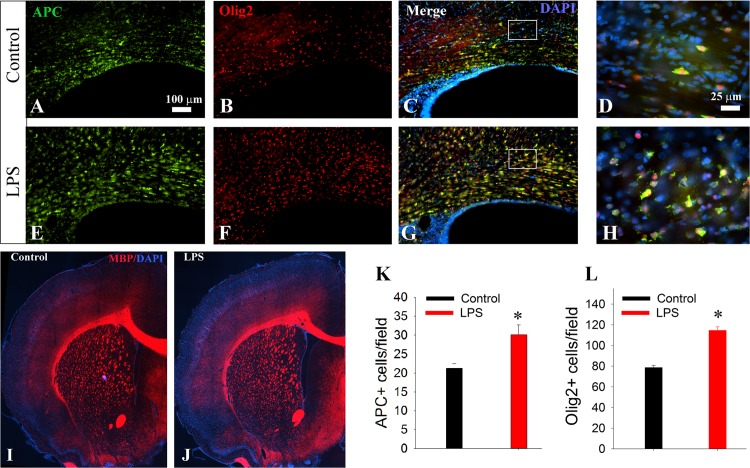
LPS exposure results in a significant increase in OLs population without affecting myelination on P21. A-H: representative micrographs show double-immunostaining of mature OLs (APC+) and total OL lineage cells (Olig2+) in the corpus callosum just above the lateral ventricle. Note a significant increased density of both APC+ and Olig2+ cells in LPS as compared to controls. D and H are high magnification micrographs taken from boxed areas in C and G, respectively. Immunostaining of MBP shows no significant difference in myelination between LPS (J) and the control (I). Cell counting in the corpus callosum showed a significant increase of APC+ mature OLs (K) and Olig2+ total OL lineages (L). *p<0.001 vs control (n = 8).

Next we assessed OLs and myelination on P21. Mature OLs, total OL lineage cells, and myelin were identified by their respective markers APC, Olig2, and MBP, respectively. Compared to the control (Fig 5A–5D), LPS treatment led to a significant increase in both APC+ mature OLs and Olig2+ total OL lineage cells (Fig 5E–5H), which was mostly prominent in the white matter areas (e.g., the corpus callosum, the cingulum, fimbria of hippocampus, fornix, internal and external capsules, etc). Cell counting in the corpus callosum revealed a 1.4- and 1.5-fold increase in APC+ (Fig 5K) and Olig2 (Fig 5L) cells, respectively. The significant increase of in APC+ mature OLs, however, did not lead to any discernable changes in myelination. MBP Immunostaining revealed rather similar pattern and intensity of myelination between LPS-treated and the control rats (Fig 5I & 5J) A significant increase in mature OLs is likely caused by overproduction of OL progenitor cells in LPS-treated rats. To test this, we examined OPCs and myelination on P12. As expected, a significant increase in PDGFR+ (a marker for OPCs) cells was observed in LPS-exposed rats. Again, no difference in myelination was noted between the control and LPS treatment (S2 Fig).

### LPS Exposure Triggers a Prolonged Cell Proliferation in the SVZ and DG

**Fig 6:**
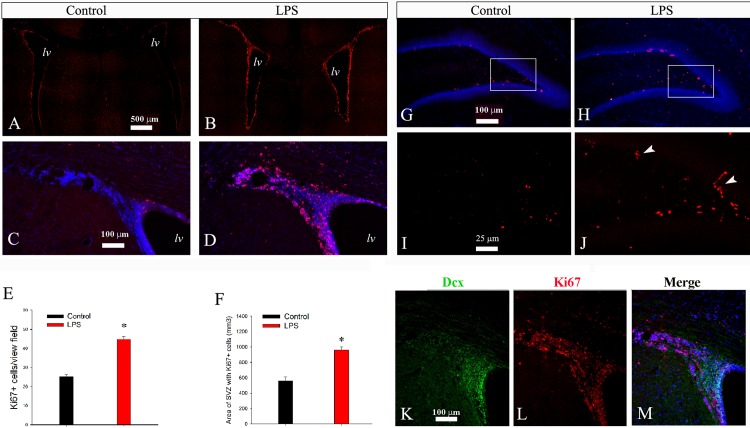
LPS exposure leads to enhanced neural cell proliferation on P21. Ki67+ cells were primarily noted in the lateral ventricles (lv) of the control (A) and LPS (B), at the striatal level at a low magnification. High power micrographs in C and D highlight ki67+ cells in the SVZ of the control and LPS treatment, respectively. Note intensively labeled ki67+ cells in the SVZ of LPS as compared to weaker immunostaining in the control, corresponding to a significant increase in both cell numbers (E) and areas (F) of Ki67 immunoreactivity in the SVZ. Similarly, a significant increase in ki67+ cells was also noted in the DG of LPS treatment (F) vs the control (E). Note clustered cells in LPS-treated (arrow heads in J), but not the control (I) animals as revealed at a higher magnification. Double-immunostaining of Dcx with Ki67 showed their partial overlap in the SVZ (K-M). Lv: lateral ventricle. *p<0.01 vs the control. (n = 8).

**Fig 7:**
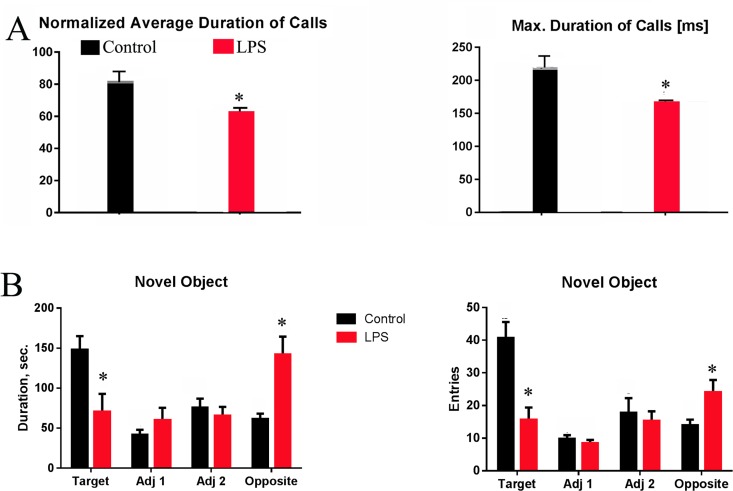
Systemic LPS-exposed rats exhibit communicative and cognitive impairments. The ultrasonic vocalization test conducted on P10 shows that LPS-exposed rats emitted a significantly reduced average calls and max duration of calls as compared to control rats (A). Test for novel object recognition on P40 shows that LPS-exposed rats spent significant shorter time duration (B, left) and fewer entries in the target corner with a novel object. Conversely, LPS-treated rats spent significantly longer time duration and more entries in the opposite corner (B, right). * p<0.05 vs control. N = 8.

The over-production of OL lineage cells as well as reduction of apoptotic cell death suggested that there might be an over-expression of growth-promoting factors following LPS exposure, raising the question whether there was an increase in neurogenesis. Therefore, we next examined cell proliferation by ki67 immunostaining on P21. As shown in Fig 6, in the control, ki67+ cells were primarily identified in the SVZ and DG, although scattered ki67+ cells were also presented in other brain regions especially the white matter. LPS treatment led to a marked increase in ki67+ cell density in both the SVZ (Fig 6A–6D) and DG (Fig 6G–6J). The intensively immunostained ki67+ cells in the SVZ tended to expand both laterally and ventrally, leading to a significant increase in the area with ki67 immunoreactivity (Fig 7F). In the DG, some ki67+ cells appeared to form clusters (arrows in Fig 6J). Double immunostaining showed that ki67+ cells in the SVZ of LPS treated animals partially overlapped with DCX, a marker for neuroblasts or immature neurons (Fig 6K–6M).

### Systemic LPS Exposure Results in Deficits in Communicative and Cognitive Functions

The overall neurobiological features following systemic LPS exposure appeared to be non-destructive (e.g. no signs of cell and tissue damage), but rather growth-promoting (e.g. decreased PCD, increased proliferation) in nature. Given that disturbances of early cell developmental milestones, such as increased cell proliferation [25], increased cortical neuronal density [26], and abnormal patterns of neuronal migration [27, 28], have been reported in ASD brains by postmortem studies, we then conducted neurobehavioral assessments to test whether ASD-like behaviors could be replicated in our animal model. Results of the ultrasonic vocalization test showed that the average as well as the maximum durations of calls emitted by pups were significantly reduced by LPS treatment (Fig 7A). The novel object recognition test showed that LPS-treated rats spent significantly shorter time duration in the target corner but more in the opposite corner, compared to control rats (Fig 7B). If the time duration for the target vs the opposite corner was compared for individual animals, control rats spent significantly longer time period in the target corner than in any other corners. In contrast, LPS-exposed rats spent significantly shorter time period in the target corners opposite to the novel object than in any other corners. This is also true for entries into corners with or without a novel object, i.e., LPS-treated rats had significantly fewer entries into the target corner than the control rats.

## Discussion

The major finding of this study is that early postnatal exposure to systemic LPS leads to a robust microglia activation characterized with mixed M1 and M2 phenotypes. The unique pattern of microglia activation is associated with a seemly growth-promoting effect on brain development and ASD-like neurobehavioral abnormalities.

Previously, we have demonstrated that intracerebral injection of LPS to P5 rats resulted in a robust proinflammatory response in the brain, as indicated by a surge of proinflammatory cytokines including TNFα, IL1β, and IL-6 in the rat brain [13], while pro-inflammatory mediators such as IL1β and iNOS were found to be co-localized with activated microglia [13, 15, 29]. In contrast, the current study revealed a mixed, M2-biased microglia polarization following systemic LPS exposure. It has been reported that in the adult animals, either intracerebral or intravenous injection of LPS induce a predominantly M1-like microglia activation [30, 31]. Thus, it appears that the differential microglial responses in the intracerebral vs systemic LPS neonatal models are specific to the developing brain. Based on the literature and our own data, several potential mechanisms may underlie this difference. First, there is a large disparity of LPS availability in the brain parenchyma between these two models. Although LPS is lipophilic, only minimal amount of LPS could cross the blood brain barrier (BBB) [31, 32]. Second, the molecular mechanisms underlying LPS-induced microglia activation following central vs systemic exposure may also be different. Intracerebral LPS is likely to activate microglia directly by activating TLR4 receptor, which is highly expressed by microglia and endothelial cells of blood vessels [33]. In contrast, due to the restriction of BBB, systemic LPS may first activate endothelial cells to release proinflammatory cytokines, which then indirectly activate microglia, although TLR4 appears to be necessary for this action [34]. Finally, there may also be intrinsic differences between immature and mature microglia, in terms of phenotypic response to LPS challenge. Although there is no direct evidence to support this hypothesis, microglia activation is known to be highly context-dependent [35, 36], while the extracellular environment is very different between the developing and mature brain.

When investigating adverse neurodevelopmental consequences of early life neuroinflammation, most studies have focused on the role of proinflammatory response and/or M1-like microglial activation in mediating neural injury and functional deficits in animals. Our data revealed that systemic LPS exposure caused a hyperplastic, rather than injurious effect on neuronal cells, which is consistent with the observation that M2 rather than M1 microglia activation dominate the early neuroinflammatory response in this particular animal model. Although many environmental insults could trigger apoptotic cell death in the developing brain, PCD is a normal developmental program that plays a pivotal role in regulating neuronal [22] and oligodendroglial [23] numbers, since both cell types are generated in excess during early developmental period. Programmed neuronal death is primarily regulated by neurotrophic factors, whereas apoptosis of OPCs is regulated by competing survival factors especially platelet-derived growth factor-AA (PDGF-AA) [37]. A marked decrease of PCD in LPS-treated rat brain may be caused by excessive production of trophic factors, most likely produced by M2-like microglia. Accumulating evidence suggest that microglia with alternatively activated phenotype play a role in adult neurogenesis. For instance, it was recently reported that there is a distinctive population of activated microglia exhibiting alternative activated phenotype in the SVZ of adult mice, as indicated by their expression of M2 cytokine profiles. Deletion of microglia in the SVZ leads to a significant decrease in both survival and migration of neuroblasts, suggesting that these alternatively activated microglia might provide trophic support for neurons [38]. This finding is consistent with in vitro evidence that IL4- activated microglia enhance, whereas IFNγ-activated microglia suppress, neurogenesis and oligodendrogenesis [39]. In the current study, we clearly demonstrated a high level of TGFβ expression in activated Iba1+ microglia, suggesting that microglial M2-associated cytokines and/or growth factors may underlie the hypertrophic effect on neuronal progenitors and OL lineage cells.

In the adult animal, newly generated neuroblasts in the SVZ and DG migrate and integrate into local circuitry. Experimental studies suggest that adult neurogenesis plays a crucial role in development of certain brain functions. For example, neurogenesis in the DG plays a significant role in acquisition of certain contextual memory functions [40]. The proliferation of neuroblasts in the SVZ and DG also showed high plasticity, since it can be enhanced under both physiological (such as physical exercise, task learning, environmental enrichment, etc.) as well as pathological (seizures, stroke, etc.) conditions [41, 42]. The effect of neuroinflammation on neurogenesis remains controversial and contradictory results have been reported. For example, Chapman et al. [43] reported increased striatal neurogenesis in the adult rat following intrastriatal LPS injection. Dinel et al. [44] demonstrated that LPS exposure at P14 leads to neurobehavioral abnormalities including altered anxiety-like and depressive-like behaviors, without affecting hippocampal neurogenesis. However, when animals at adulthood were challenged with a second LPS, there was a significant decrease in hippocampal neurogenesis. It is worth noting that ages of animals, routes of LPS administration, and the time course between LPS treatment and neurogenesis assessment, are different between these studies and ours. Given that microglia respond to immune challenge in a highly context-dependent manner, the neurobiological mechanisms underlying LPS-mediated effect on neurogenesis reported in those studies might be very different. A more comparable experimental setting from this study is the work by Smith et al.[45], who reported that LPS exposure at P5 does not affect total numbers of Brdu+ cells in the hippocampus at P8, P21 and P74 in mice. However, both type 3 neuronal precursors and Brdu+/Dcx+ double-labeled cells were reduced, suggesting an inhibitory effect of LPS on proliferating neuroblasts. A possible contributing factor for the different effect of LPS on neurogenesis observed between our study and by Smith et al. might be different approaches used to label proliferating cells. In their study, Brdu was injected 24 h before animals were sacrificed (P21 and P74), thus Brdu positive cells represent all cells at S phase during this narrow window. The ki67 labeled cells in our study comprise of all cells in the proliferating cycle except the resting phase. It was demonstrated that the number of Ki67+ cells are about 50% higher than Brud labeled cells [46]. In addition, different animal species (rats vs mice) and the timing of LPS administration (P3 vs P5) may be other potential contributing factors. In brief, the effect of neuroinflammation on developmental neurogenesis is inconclusive and future studies are needed to clarify this issue.

Compared to adult neurogenesis, the functional significance of enhanced neurogenesis/gliogenesis during perinatal and neonatal periods, remains to be elucidated. From a developmental standpoint, many early neural development programs are highly orchestrated in a spatiotemporally coordinated manner so that the right number of neurons and synapses, and the precise circuitry and network can be established. Thus, one can readily speculate that any deviations (either increase or decrease) of cell development programs can be detrimental to neural network maturation, which may be reflected by altered neurobehavioral abnormalities. In fact, studies suggest that many of the early cell development events are altered in ASD. For example, accelerated brain overgrowth at 1–5 years of age is a defining neuroanatomical feature of ASD [47], which might reflect overproduction of neural cells. This is supported by several postmortem ASD studies reporting increased cortical neuron density [26, 48], cell proliferation [25], abnormal pattern of neuronal migration [27], as well as altered GABAergic neurons [49] indicative of abnormal neuronal differentiation. As mentioned earlier, reduced apoptosis might be a result of excessive anti-inflammatory cytokines and/or growth factors released from M2 polarized microglia. The observation that there was not only an increased cell proliferation in the neurogenic niches but also overproduction of OL population in the white matter is in line with this notion. Additional evidence to further support this hypothesis is that we also observed a significant increase in OPCs on P12. OPCs are known to rely on growth factors especially the platelet derived growth factor (PDGF) and basic fibroblast growth factor (bFGF) for survival and proliferation [50].

The communicative and cognitive deficits in LPS-exposed rats suggest that early postnatal systemic LPS exposure could lead to ASD-like symptoms. Currently, ASD diagnosis is based purely on behavioral criteria, with language and communicative impairment at the core of behavioral deficits. In addition, the majority of ASD patients are also cognitively impaired. In animal studies, ASD-like behavioral impairments were most commonly replicated in maternal LPS or poly I:C models [7]. To the best of our knowledge, this is the first study to demonstrate that early postnatal LPS exposure leads to a M2-biased microglia polarization and ASD-like behavioral impairments in animals. Although cell developmental abnormalities observed in this animal model have also been reported in human ASD studies, the underlying mechanisms remain elusive. Given that there is overwhelming evidence suggesting a dysregulated immune system as well as microglia abnormalities in ASD [51], a linkage between dysregulated microglia activation and early neural development is plausible. Critically, a recent human study strongly suggests this possibility. In analyzing postmortem cortical tissue, Gupta et al. demonstrated that many myelin related genes and microglial M2 genes were significantly augmented in ASD patients compared to controls. Remarkably, the upregulated M2 microglia gene module was negatively correlated with a differentially expressed neuronal gene module, suggesting a causative role of dysregulated M2 microglia activation and aberrant neuronal development [52].

Although we observed some similarities between the current animal model and ASD in terms of cell development and behavioral phenotype, this postnatal LPS treatment is by no means to be an ideal animal model for ASD. In fact, the maternal immune activation (MIA) model is more commonly used in ASD study, due to both good face (behavioral phenotypes) and construct (similar cause) validity. Nevertheless, the current model still holds value in studying biological mechanisms underlying aberrant early cell development by inflammatory challenge, some of which may be shared across multiple neurodevelopmental disorders including ASD, schizophrenia, and depression.

## Supporting Information

S1 Fig Morphological profiles indicate that microglia in the LPS-exposed rats were not full resolved on P21. Microglia were immunostained with Iba1 antibody to reveal their morphology. Representative images taken from coronary sections at mid-hippocampal level show all layers of the cerebral cortex, white matter, and hippocampus of the control (panel A) and LPS-treated animals (D). Regardless of treatment, the morphology of microglia was quite heterogeneous across different brain regions, with more ramified cells seen in the cerebral cortex (showing layer 1, 4, and 6) while less-ramified cells seen in other regions such as the (WM), CA area and DG of hippocampus. However, when compared in a regional specific manner, it becomes apparent that microglia in the LPS group had fewer processes and bigger stomata, in comparison with the controls. Panel B&E show images taken from indicated regions (red arrows) of control or LPS group by 20× objectives. C&F further highlight individual cells from corresponding images in panel B&E, with higher magnifications.
(TIF)

S2 Fig LPS-exposure led to a significant increase in OPC population on P12. OPCs were identified by PDGFR (A-D) and myelin was revealed by MBP immunostaining (F&G). LPS treatment caused a significant increase in OPC population (E), as shown in both the cortex (A&B) and corpus callosum (C-D). However, no discernable difference in myelination was noted between the control (F) and LPS (G) treatment. *p<0.01 vs controls (n = 4).
(TIF)

## Abbreviations

LPS: lipopolysaccharide
OL: oligodendrocyte
OPC: oligodendrocyte progenitor cells
PCD: programmed cell death
WMI: white matter injury
ASD: Autism Spectrum Disorders
CNS: central nervous system
TGFβ: transforming growth factor-beta
TUNEL: Terminal deoxynucleotidyl transferase mediated dUTP nick end labeling
MBP: myelin basic protein
MHC-2: major histocompatibility complex-2
iNOS: inducible nitric oxide synthase
Iba1: ionized calcium-binding adapter molecule
