# Supplementary figures and images for: Early Postnatal Lipopolysaccharide Exposure Leads to Enhanced Neurogenesis and Impaired Communicative Functions in Rats
Source: PLoS One. 2016 Oct 10;11(10):e0164403. doi: 10.1371/journal.pone.0164403 (PMC5056722; doi:10.1371/journal.pone.0164403)

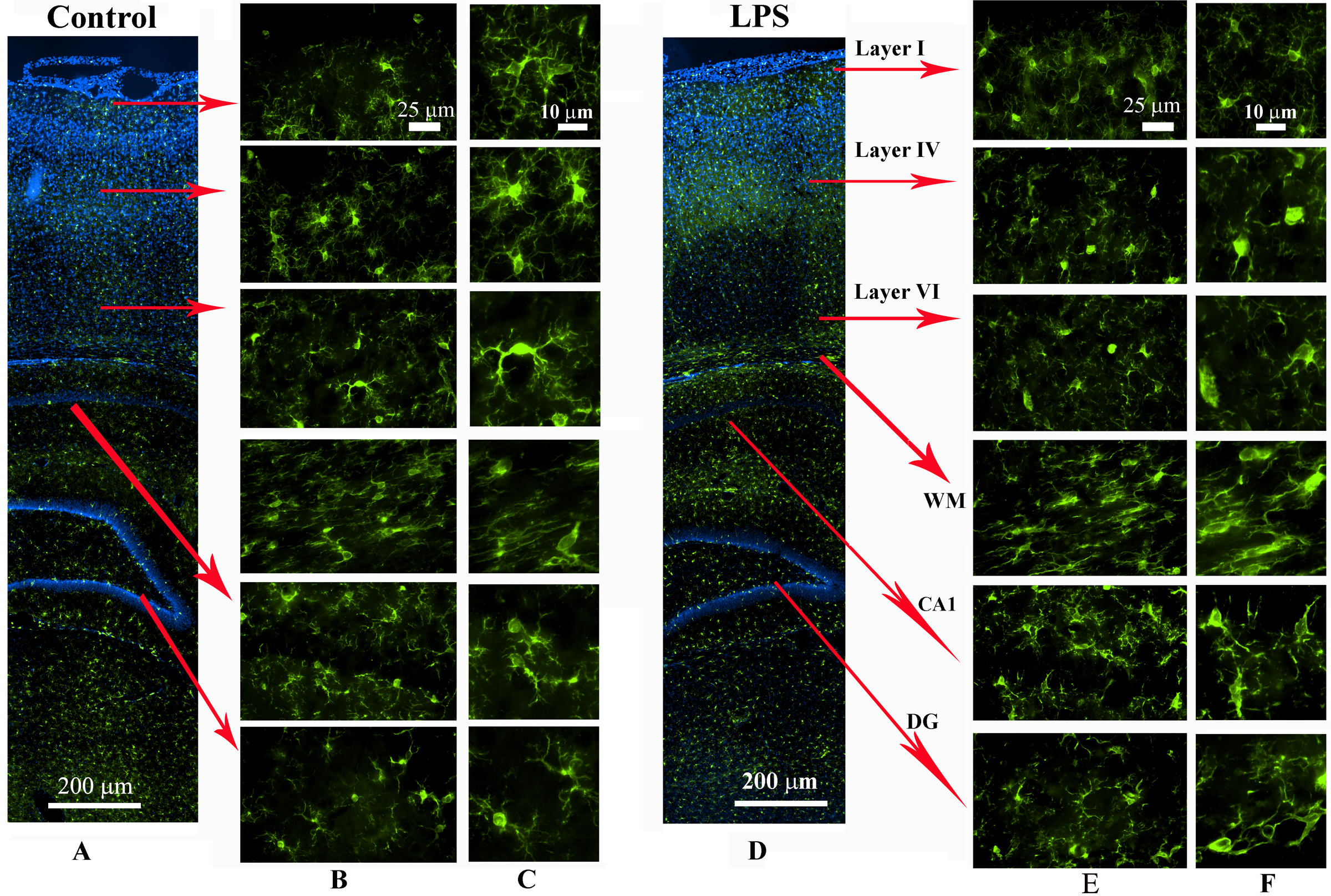

Supplement: S1 Fig — Microglia were immunostained with Iba1 antibody to reveal their morphology. Representative images taken from coronary sections at mid-hippocampal level show all layers of the cerebral cortex, white matter, and hippocampus of the control (panel A) and LPS-treated animals (D). Regardless of treatment, the morphology of microglia was quite heterogeneous across different brain regions, with more ramified cells seen in the cerebral cortex (showing layer 1, 4, and 6) while less-ramified cells seen in other regions such as the (WM), CA area and DG of hippocampus. However, when compared in a regional specific manner, it becomes apparent that microglia in the LPS group had fewer processes and bigger stomata, in comparison with the controls. Panel B&E show images taken from indicated regions (red arrows) of control or LPS group by 20× objectives. C&F further highlight individual cells from corresponding images in panel B&E, with higher magnifications. (TIF) [file pone.0164403.s001.tif]

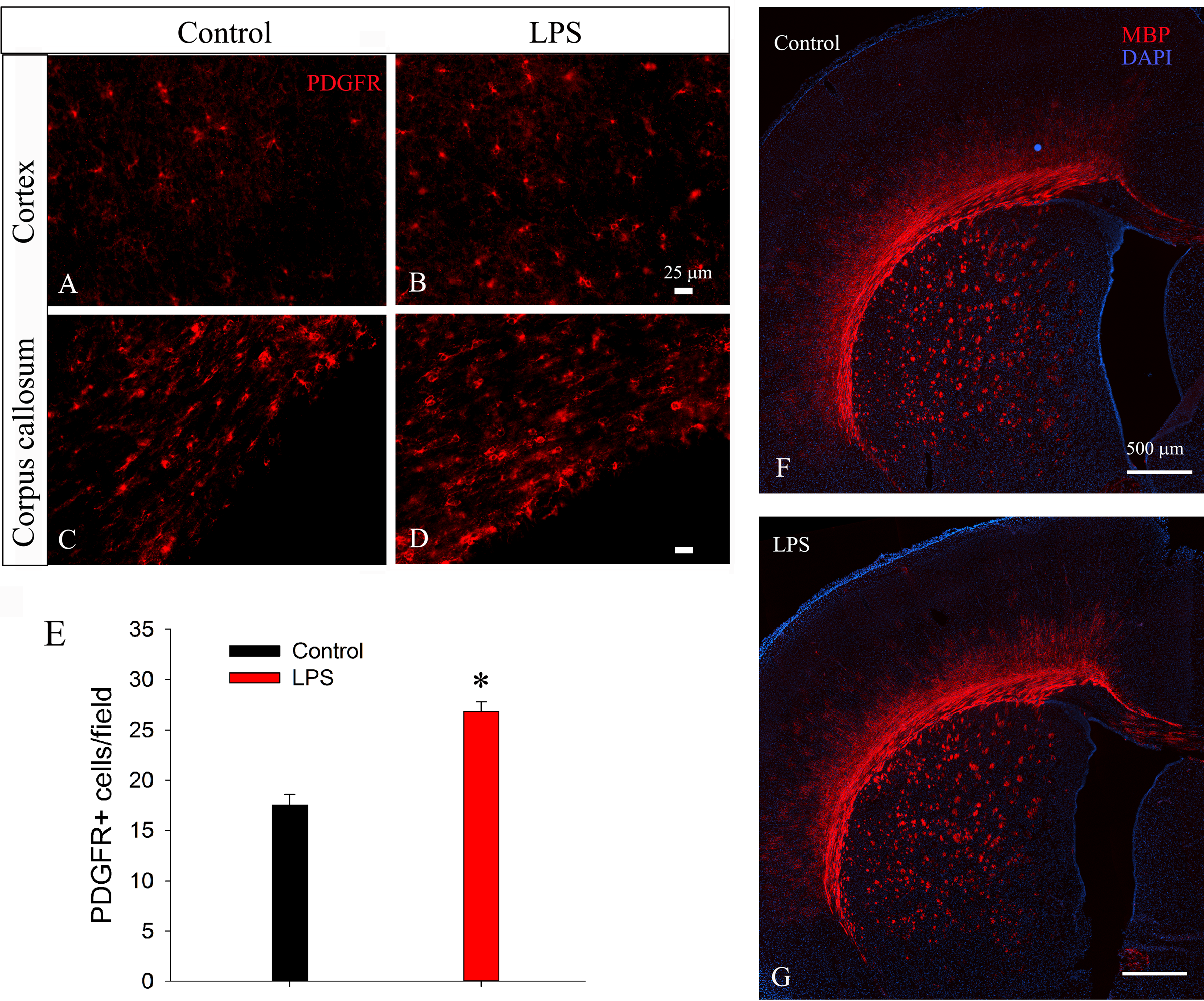

Supplement: S2 Fig — OPCs were identified by PDGFR (A-D) and myelin was revealed by MBP immunostaining (F&G). LPS treatment caused a significant increase in OPC population (E), as shown in both the cortex (A&B) and corpus callosum (C-D). However, no discernable difference in myelination was noted between the control (F) and LPS (G) treatment. *p<0.01 vs controls (n = 4). (TIF) [file pone.0164403.s002.tif]
